# Aging of the mammalian gastrointestinal tract: a complex organ system

**DOI:** 10.1007/s11357-013-9603-2

**Published:** 2013-12-20

**Authors:** M. Jill Saffrey

**Affiliations:** Department of Life Health and Chemical Sciences, Biomedical Research Network, The Open University, Milton Keynes, MK7 6AA UK

**Keywords:** Enteric nervous system, Interstitial cells, Intestinal smooth muscle, Intestinal immune system, Mucosal epithelium, Stem cells, Diet, Microbiota

## Abstract

Gastrointestinal disorders are a major cause of morbidity in the elderly population. The gastrointestinal tract is the most complex organ system; its diverse cells perform a range of functions essential to life, not only secretion, digestion, absorption and excretion, but also, very importantly, defence. The gastrointestinal tract acts not only as a barrier to harmful materials and pathogens but also contains the vast number of beneficial bacterial populations that make up the microbiota. Communication between the cells of the gastrointestinal tract and the central nervous and endocrine systems modifies behaviour; the organisms of the microbiota also contribute to this brain–gut–enteric microbiota axis. Age-related physiological changes in the gut are not only common, but also variable, and likely to be influenced by external factors as well as intrinsic aging of the cells involved. The cellular and molecular changes exhibited by the aging gut cells also vary. Aging intestinal smooth muscle cells exhibit a number of changes in the signalling pathways that regulate contraction. There is some evidence for age-associated degeneration of neurons and glia of the enteric nervous system, although enteric neuronal losses are likely not to be nearly as extensive as previously believed. Aging enteric neurons have been shown to exhibit a senescence-associated phenotype. Epithelial stem cells exhibit increased mitochondrial mutation in aging that affects their progeny in the mucosal epithelium. Changes to the microbiota and intestinal immune system during aging are likely to contribute to wider aging of the organism and are increasingly important areas of analysis. How changes of the different cell types of the gut during aging affect the numerous cellular interactions that are essential for normal gut functions will be important areas for future aging research.

## Introduction: aging of a complex system

The mammalian gastrointestinal (GI) tract is unique among the organ systems. It comprises anatomically and functionally distinct regions and consists of diverse cell types; a diversity that is unmatched in other organ systems. It contains the largest number and most complex system of neurons outside the central nervous system, the largest population of immune system cells in the body and a diversity of specialised epithelial cells. Although the different types of cells in the gut have distinct functions, the interactions between them play a key role in the regulation of normal gut physiology. These cellular interactions are now appreciated to be much more complex than previously believed.

In addition to its intrinsic cellular complexity, the GI tract is host to the enteric microbiota: a vast, diverse and varying population that is now increasingly understood to interact with the gut and indeed the whole organism in a number of critical ways. The GI tract is also closely associated with other organ systems. These include glandular organs (the liver, pancreas, gall bladder and salivary glands) and also autonomic and sensory neurons and the vasculature, all of which play essential roles in specialised gut functions. GI functions are thus the result of integrated activities of an enormous range of cell types. Changes in any of these cells during aging may therefore profoundly influence GI functions.

Aging is associated with a number of GI disorders, some of which have a major impact on the quality of life of affected individuals. Other seemingly non-GI age-associated disorders may also be related to aging of cells of the gut. For example, impairment of the intestinal immune system as a result of aging is likely to be a major factor in the increase in the incidence and severity of infections in the elderly. Understanding how the cells of this organ system change during aging is thus a critically important area for gerontology research. In this review current understanding of aging of the cells and tissues of the mammalian GI tract is discussed. The focus is on cellular aging, although physiological changes are also briefly described. First, the changes in gut function that occur in the aging human population are considered.

## Gastrointestinal function is compromised during aging

Disorders of the GI tract are prevalent amongst the elderly population. In addition to an increased incidence of GI cancers, which are not discussed here, disorders such as dysphagia, reflux, chronic constipation, fecal impaction and incontinence are common; while delayed gastric emptying and impaired absorption, often linked to bacterial overgrowth, have also been described in some studies (see Firth and Prather 2002). The intestinal immune system is impaired in aging; the elderly show increased susceptibility to gut infections (see (Ogra 2010) and intestinal inflammation also increases with advancing age. An additional age-related condition that involves the GI tract is anorexia of aging (Moss et al. 2012). Many of these individual conditions, in addition to causing specific local symptoms within the gut and its associated organs, can contribute to malnutrition, which is common and results in increased frailty and vulnerability of many elderly people.

The incidence of some GI disorders in the aging population is striking. For example, more than 50 % of elderly people in care homes experience chronic constipation; up to 74 % of this group use laxatives on a daily basis (see Gallagher and O'Mahony 2009). Among adults living in the community, the incidence of chronic constipation is around 15 % in the total population, but increases to 30–40 % among those over 65 years of age. Chronic constipation is very commonly associated with fecal impaction, and perhaps most distressingly, also with fecal incontinence, which is reported to affect around 5 % of individuals over 65 years in the general community (although some estimates indicate that as many as 30 % report occasional incontinence) but some 50 % of those in care homes (see Chatoor et al. 2007; Stevens et al. 2003). The costs of these and other GI disorders, in terms of both quality of life and healthcare expenditure, are very considerable indeed.

The causes underlying the increased incidence of GI conditions in older people are only poorly understood. In addition to aging of the cells of the GI tract, comorbidity, medication and decreased mobility are also likely to be involved in the etiology of GI disorders in the elderly. Thus, the causes of GI dysfunction in the elderly are likely to be complex and multifactorial. Evidence that other extrinsic factors, such as exercise, diet and the microbiota, may influence both GI function and cellular properties during aging is discussed in a later section of this review.

The analysis of changes in the GI system during aging has been performed at many levels, for example whole organism studies of food intake and stool frequency, organ studies such as measurement of movement of contents along the GI tract (or along specific regions), physiological and pharmacological analysis of isolated samples, electrophysiological, cellular and, in a few cases, molecular analyses. The results of these studies will be considered after a brief summary of the organisation of the GI tract and its component cells.

## A brief overview of the cellular organisation and functions of the mammalian gastrointestinal tract

The GI tract extends from the mouth to the anus and is associated with other organs including the liver, pancreas and gall bladder. The main subdivisions of the GI tract are the oesophagus, stomach, and the small and large intestines. However, functionally specialised regions exist within these subdivisions (e.g. the duodenum, jejunum and ileum of the small intestine) and sphincters play an essential role in regulating the passage of gut contents between the main functionally distinct areas of the GI tract.

**Fig. 1:**
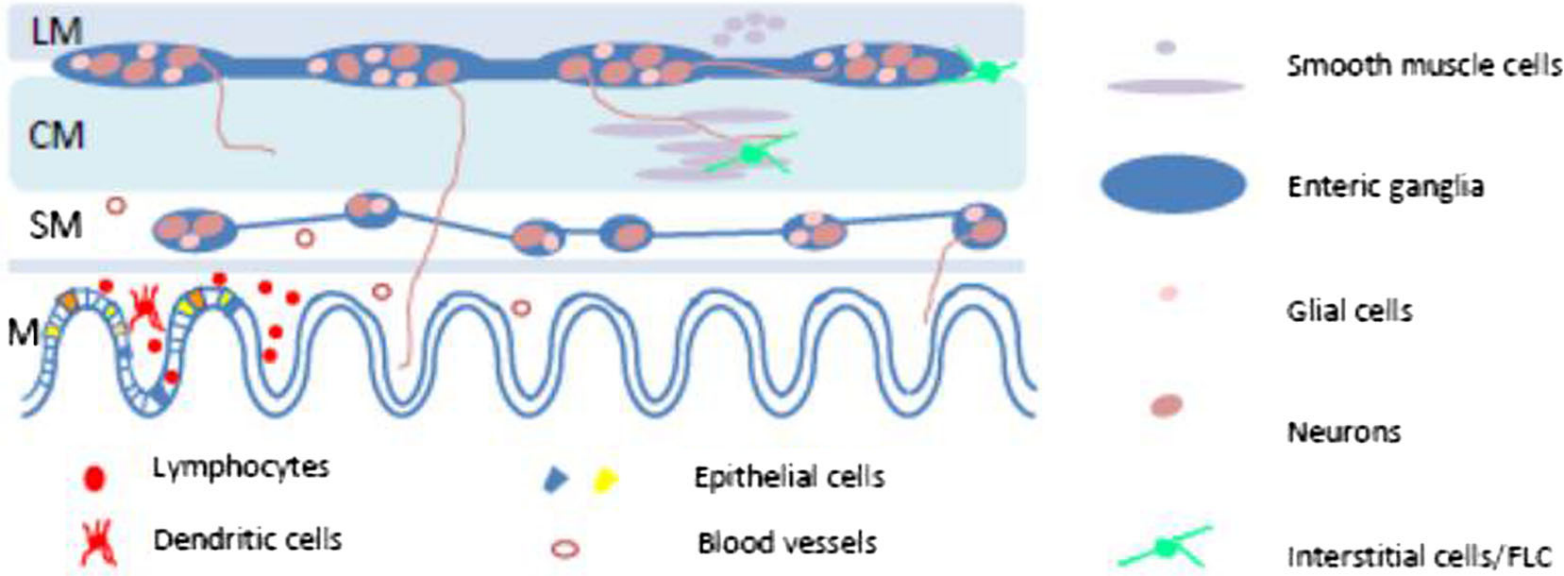
Simplified schematic diagram showing the main tissue layers and cell types of the gastrointestinal tract. *LM* longitudinal muscle, *CM* circular muscle, *SM* submucosa, *M* mucosa

The wall of the GI tract is organised into three main tissue layers, although additional layers are present in some regions and species. Each of these layers is composed of a number of different types of cell (Fig. 1). The arrangement of the different layers of the gut wall and their component cells is broadly similar along the entire length of the gut, but there are some differences, for example, in the arrangement of the epithelium and the thickness of the smooth muscle in different gut regions. An example is the specialised glandular regions of the stomach.

Gut functions, such as digestion, absorption, movement of gut contents and defecation, are the result of coordinated actions of diverse gut cells, including smooth muscle cells, intrinsic neurons and epithelial cells of several different types (including absorptive enterocytes, the acid-secreting parietal and pepsinogen-secreting cells in the stomach, mucus-secreting goblet cells and intestinal endocrine (enteroendocrine) cells). However, other cells within the gut wall, notably specialised interstitial cells and enteric glial cells, also play key roles in gut functions and the vast and diverse population of cells that comprise the mucosal immune system play a vital role in defence. Finally, communication between the gut and the brain via extrinsic autonomic (vagal and sacral) and sensory nerves and the hormones produced by both enteroendocrine and other endocrine cells is of major importance. This communication, via what is known as the brain–gut axis, is bi-directional; not only do extrinsic signals regulate gut functions, but information conveyed from the gut via enteric neurons and extrinsic sensory neurons and enteroendocrine cells also influences some central nervous system activities that impact upon behaviour, including appetite regulation (Sam et al. 2012). Most recently, the microbiota have been realised to impact upon these processes; the brain–gut–enteric microbiota axis is currently an area of intense research (Cryan and O'Mahony 2011; Forsythe and Kunze 2013; Rhee et al. 2009).

## Animal models in the study of GI aging

Age-related changes in GI function in humans are difficult to analyse not only for ethical reasons, but also because of the many other factors that can influence GI physiology (see above). In order to avoid these confounding variables, animal models have been used to investigate changes in GI functions during aging. However, despite the fact that young adult animals, particularly guinea-pigs, but also rats and more recently mice, have been very extensively used in basic research on GI physiology, relatively few physiological studies on samples from aging animals have been performed. Data from such studies indicate that age-associated changes do occur, but the results from different studies have not always been consistent. Similarly, the more numerous morphological and cellular studies of the aging gut in animal models have often produced conflicting data.

There are a number of reasons why results from different animal studies may vary. In addition to differences in the detail of the techniques used, differences between strains of animals and regions of the gut used and issues of husbandry are likely to contribute to inter-study variation. One important feature of the GI tract that can affect many types of investigation into GI aging is that its size changes during the lifespan; it continues to grow well past ‘middle age’ (e.g. Gabella 1989; Peck et al. 2009; Phillips and Powley 2001; see Saffrey 2013). Thus, standardisation of some types of results according to muscle thickness, or mucosal area, for example, is necessary. These issues are discussed more fully elsewhere (Kapur 2013; Phillips and Powley 2007; Saffrey 2013).

A fundamental but also crucial question concerning the use of animal models for the study of GI aging is whether or not the animals used display similar *general* GI functional changes to those that occur in many elderly people. For example, if the animals used for aging studies do not exhibit changes such as increased colonic transit time, decreased stool formation, and their patterns of defecation are normal, it could be argued that they are not a good model for the study of the aging human gut. Surprisingly few studies have addressed this issue, even in cohorts of animals that have been used in standard organ bath physiology or for analysis of cellular changes (described below). However, delayed gastric emptying, increased colonic transit time and decreased stool production have been reported in aging rats (Smits and Lefebvre 1996), and recent work has shown that reduced fecal pellet production and delayed colonic transit also occur in aging C57BL/6 mice (Patel et al. 2012). Thus, some important age-related general functional changes in the stomach, large intestine and terminal bowel, in at least some animal models of aging, appear to be similar to those observed in humans.

The study of specific functional changes in different cell types during aging has involved ex vivo analysis of gut samples to examine parameters such as absorption, ion transport and smooth muscle contractility, and responses to nerve stimulation (and/or the addition of agonists and antagonists of the neurotransmitters and modulators involved in normal GI reflexes). Some studies of isolated cells have also been performed.

## Aging of intestinal smooth muscle

Intestinal smooth muscle activity is fundamental to GI function. There are several different types of gut movements, which vary in different GI regions. For example, in the stomach two different types of smooth muscle movements mix ingested food with gastric secretions and are responsible for gastric emptying. In the intestine, different types of movement include the peristaltic reflex, which involves both relaxation and contraction of smooth muscle in response to the presence of a bolus in the gut lumen, and also regular rhythmic or waves of contraction such as the colonic migrating motor complex The contraction and relaxation of intestinal smooth muscle are regulated by enteric nerves and by specialised interstitial cells (see Sanders et al. 2010; interstitial cells of Cajal (ICC) and similar fibroblast-like cells (FLC, also known as PDGFR-α-positive cells).

Although evidence suggests that motility is impaired in some GI regions during aging, whether this is due to impaired function of the smooth muscle cells themselves or to disruption of the cells that regulate their activity (neurons, ICCs and FLCs), or both, is difficult to determine. Studies of the responses of smooth muscle in isolated gut samples to electrically induced nerve stimulation indicate that smooth muscle contractility may be increased in older animals; however, this could, in part at least, be due to the increased thickness of the muscle layers seen in older animals (see Hoyle and Saffrey 2012). Changes in the responses of such samples to applied neurotransmitters or agonists in old animals have produced varying results. For example, some studies have shown increased contractile responses to cholinergic agonists, while others have shown decreased responses or no change.

A number of changes in the cellular proteins and mechanisms that regulate the contractile properties of intestinal smooth muscle in aging have been described by Bitar (see (Bitar 2003, 2005; Bitar and Patil 2004). Changes in the signal transduction pathways that regulate the phosphorylation of myosin light chain have been described in the smooth muscle of aging rat colon (see Bitar 2003; Bitar and Patil 2004). Among other changes in these pathways, the translocation of Rho A and protein kinase C (PKC)-α to caveolae at the smooth muscle cell membrane has been shown to be reduced in aging muscle (Somara et al. 2007). The expression of caveolin 1 was also shown to be reduced in aging rat colonic smooth muscle (Somara et al. 2007), and there was an impaired movement of caveolin 1 in response to application of acetylcholine (Somara et al. 2011). The reasons why these changes in key proteins occur during aging, however, are not clear.

Calcium signalling may also be dysregulated in aging intestinal smooth muscle. A decrease in calcium channel currents and intracellular Ca^2+^ levels has been described in isolated rat colonic smooth muscle cells during aging (Xiong et al. 1993), while increased calcium stores in mitochondria and the sarcoplasmic reticulum have also been described in aging rat colon smooth muscle (Lopes et al. 2006).

Morphological evidence for degenerative changes in the colonic smooth muscle during aging has also been presented. Abnormalities of mitochondrial structure and apoptosis of smooth muscle cells have been described in the colonic muscle of old animals (Lopes et al. 2004). Evidence for impaired mitochondrial function in aging smooth muscle was also described by these authors.

## Aging of intestinal interstitial cells

Interstitial cells, which are located within the smooth muscle and also around the enteric ganglia, are difficult to quantify because of their small cell bodies and long, fine processes that form a network-like arrangement. Only one study to date has examined changes of these cells in aging. Gomez-Pinilla et al. (2011) have reported a reduction in the number and network volume of interstitial cells in the aging human stomach and colon.

## Aging of the cells of the enteric nervous system

The enteric nervous system (ENS) is the largest part of the peripheral nervous system in terms of neuron numbers, and it is also the most complex, comprising some 16 functionally distinct subpopulations of neurons (see Furness 2006, 2012). Enteric neurons are grouped into networks of small ganglia, which are present along the length of the GI tract. Enteric ganglia are, in most regions, irregular in both size and shape and contain a mixture of the functionally distinct neuronal subtypes. Enteric neurons play an essential role in the regulation of GI functions; they are responsible for the coordination of activities of other GI cells, thus influencing processes such as gut movements (motility) absorption and secretion and the microvasculature. Recent evidence indicates that enteric neurons also influence epithelial barrier functions (e.g. Neunlist et al. 2013). The great majority of, but not all, activities of the ENS occur independently of extrinsic innervation.

### Myenteric neurons

Research on ENS aging has largely focused on analysis of changes in neuronal numbers in the myenteric plexus, which has been studied in a number of species, including humans (see Phillips and Powley 2007; Saffrey 2013). Many such studies have reported a loss of myenteric neurons during aging, which in some cases was as much as 50–60 % (Cowen et al. 2000; Santer and Baker 1988), but other more recent studies have reported that there is no significant myenteric neuronal loss with increasing age (Gamage et al. 2013; Peck et al. 2009; Van Ginneken et al. 2011). Moreover, in those studies that do describe a reduction in the number of neurons in the gut of old animals, the level of loss is highly variable (see Saffrey 2013).

One of the reasons for the discrepancies between the results of different studies of neuronal loss in the ENS may be technical, brought about by the difficulty of accurately quantifying neuronal numbers in complex networks in an organ that undergoes changes in size during the lifespan (see Phillips and Powley 2007; Saffrey 2013). Other factors, however, may also be involved. For example in the case of animal studies, housing (e.g. the numbers of animals that are co-housed and/or environmental enrichment), diet and the microbiota may influence cellular aging in the GI tract. Very few studies have investigated the effects of diet on GI aging, but where it has been studied, caloric restriction has been reported to reduce (da Silva Porto et al. 2012; Thrasivoulou et al. 2006) or eliminate (Cowen et al. 2000; Johnson et al. 1998) the neuronal loss in Sprague–Dawley rats (and see below).

The time course of reported reductions in myenteric neuronal numbers has been studied in some cases, although some studies just report the differences between ‘young’ and ‘old’ animals. Interestingly, differences in the time of onset and/or completion of reductions in neuronal numbers have been described in different studies. For example, in Sprague–Dawley rats, reductions have been reported to start at 13 months and be complete by 16 months (Cowen et al. 2000), while in Fischer 344 rats, losses have been reported to start between 6 and 12 months, but continue to 21 or more months of age (Phillips and Powley 2007). These differences highlight both possible strain differences, and also the possible effects of husbandry on aging of the GI tract. Importantly, the natural lifespan of the different species and strains of animals used in aging gut studies has been little considered, but could clearly influence the interpretation of data from aging GI studies. This is just one example indicating that careful analysis of the reasons for differences between the extent of neuronal loss in different studies may reveal important information, leading to a better understanding of the factors that cause/accelerate neuronal aging in the ENS. A full discussion of the issues involved in the study of change in enteric neuronal numbers during aging is given by Phillips and Powley (2007) and Saffrey (2013).

While the extent of enteric neuronal loss during aging is thus controversial and likely to be variable in the ENS, there is other evidence for age-related enteric neurodegeneration. Swollen and dystrophic nerve fibres have been described in the aging rat (Phillips et al. 2003) and mouse (Gamage et al. 2013) gut by light microscopy. Electron microscopy has also shown degenerating nerve fibres in aging rat small intestine (Feher and Penzes 1987). Accumulation of lipofuscin has been described in aging enteric neurons in both rats (Corns et al. 2002) (Phillips et al. 2004) and guinea-pigs (Abalo et al. 2005, 2007). Further evidence for enteric neurodegeneration during aging comes from recent studies that have shown α-synuclein-immunoreactive aggregates and, in the proximal small intestine, hyper-phosphorlylated Tau in some myenteric neurons in aging Fischer 344 rats (Phillips et al. 2009).

An important issue in ENS aging is that different neuronal subpopulations may be differentially affected. Two major groups of myenteric neurons are cholinergic neurons, which are excitatory, and nitrergic neurons, which are inhibitory. Within each of these major groups, however, are functionally distinct subgroups, for example cholinergic neurons include smooth muscle motor neurons, interneurons and intrinsic sensory neurons (see Furness 2006, 2012). Subpopulations of neurons may exhibit differential vulnerability to age-related damage or loss; several studies have provided evidence that cholinergic neurons are reduced in number in older animals, while nitrergic neurons have been reported to be spared (Cowen et al. 2000; Phillips et al. 2003). However, there are several subpopulations of cholinergic neurons and it is currently not clear which types may be affected.

In addition to loss and/or degenerative neuronal changes, other changes have been reported in the aging ENS. For example, reactive oxygen species (ROS) have been shown to be higher in myenteric neurons in the ileum of old rats than in those of young animals (Thrasivoulou et al. 2006). Aging myenteric neurons also display a senescence-associated phenotype (Jurk et al. 2012 and see section on mechanisms of aging in the GI tract, below).

### Submucosal neurons

Analysis of changes in the number of submucosal neurons during aging has been described in only three studies, on three different species. Phillips et al. (2007) described a reduction in submucosal cell numbers in different regions of the rat gut. This reduction was first detected in animals at 12 months of age compared to 6-month-old controls and continued in a linear fashion through 27 months (the oldest age examined). Reduction in submucosal neuron numbers has also been reported in mouse gut (El-Salhy et al. 1999). No change in the numbers of submucosal neurons, however, was found in a study of human gut (Bernard et al. 2009).

### Changes in nerve fibre density in the gut during aging

An expected effect of neuronal loss or degeneration in the aging gut is that there would be a reduction in the density of nerve fibres, for example in the muscle or mucosal layers, which may have functional implications. Few studies have investigated whether there is a change in the density of enteric nerve fibres in the smooth muscle or other layers of the GI tract. Peck et al. (2009) found a decrease in neuronal nitric oxide synthase- and substance P-immunoreactive nerve fibre density in the aging guinea-pig colon smooth muscle, and Wang et al. (2013) found a decrease in the areal density of immunoreactivity for the same markers in the internal anal sphincter muscle of aging mouse. A reduction in the density of these and also VIP immunoreactivity was also measured in the mucosa in that study. A problem with the analysis of fibre density by immunohistochemical techniques, however, is that an apparent reduction in fibre density may in fact reflect a reduction in the expression of the mediators being studied. Although such a change could affect function, it is also possible that sufficient levels are present for function to be retained. This problem highlights the importance of a multi-level approach to the analysis of age-related changes not just in the GI tract but in all systems.

### Changes in enteric neuronal function during aging

Most analysis of changes in neuronal function during aging has been performed by assessing the effects of nerve stimulation on target cells in ex vivo samples from animals of different ages. These preparations include a range of different cells, so discrimination between effects of aging on neuronal and target cell (e.g. smooth muscle cell) properties can be difficult and to date this issue has not been clearly resolved. Direct analysis of changes in neuronal properties during aging, such as study of the electrophysiological characteristics of different types of enteric neurons, has not been performed.

### Enteric glial cells

Changes in enteric glial cells during aging have also been little-studied. Enteric glial cells are different from the satellite cells of other autonomic ganglia and from Schwann cells, but share some characteristics with astrocytes of the central nervous system (see Ruhl 2005). They are present within the enteric ganglia and are also associated with nerve fibres in the numerous small fibre bundles that innervate the smooth muscle, submucosa and mucosa. Recently, enteric glia (and neurons) have been shown to have roles in intestinal epithelial barrier function (see Neunlist et al. 2013; Savidge et al. 2007 and below).

A reduction in the number of enteric glial cells within the myenteric ganglia of both small and large intestines (apart from the rectum) of aged rats has been described (Phillips et al. 2004). This reduction in glial cell number was found to be proportional to that of myenteric neurons. The time course of the loss was not established, however. A reduction in glial cell numbers during aging is of interest, because these cells are not post-mitotic, and new glia can be formed from precursors in adult animals (Joseph et al. 2011 and see below). Hence, a loss of glia in aging may indicate that there is an age-related deficit either in neural stem cells or the mechanisms that regulate their differentiation into glial cells.

### Aging of enteric neural crest-derived stem cells

The presence of a population of neural crest-derived stem cells in the adult enteric nervous system is now established (Kruger et al. 2002; Metzger 2010). Enteric glial cells from adult animals have also recently been shown to have the potential to differentiate into neurons in cell culture (Joseph et al. 2011; Laranjeira et al. 2011). While both neural crest-derived stem cells and enteric glia have the ability to generate neurons and glia in vitro, to date there is no evidence for the generation of new enteric neurons in the adult gut, except after injury (see Gershon 2011; Metzger 2010). Whether or not neural crest-derived stem cells and/or enteric glia retain the ability to generate new neurons and glia in the aging gut remains to be determined. Given the dispersed nature of the ENS, this question is not a trivial one to answer, and it may well be possible that areas of neural regeneration occur in response to local injury.

## Aging of the intestinal mucosa

### Diverse functions of the intestinal mucosa

The intestinal mucosa is a highly complex tissue. In addition to its well-known functions of absorption and secretion, it contains hormone-producing cells, blood vessels, many neuronal processes with associated glial cells and a vast population of immune system cells. It has two main components, the epithelium and the underlying connective tissue (the lamina propria), which are structurally different but functionally interdependent.

The intestinal epithelium is not only the site of absorption and secretion, but it also presents a barrier to damage from acid, digestive enzymes, microbes and from potentially harmful ingested material, such as some medication, an example being non-steroidal anti-inflammatory drugs. The barrier is formed not only by the tight junctions between epithelial cells, but also by mucus, bicarbonate and anti-microbial peptides secreted from specialised epithelial cells (Paneth cells). The secretion of mucus is stimulated by some gut hormones such as gastrin, which are secreted by the enteroendocrine cells, by prostaglandin and possibly also by enteric nerves. Recent evidence indicates that cells of the ENS also influence epithelial barrier function (Neunlist et al. 2013; Savidge et al. 2007).

The intestinal epithelium also contains a vital population of specialised epithelial cells with a key role in defence; these are the M cells (microfold or membranous cells because of the infolding of their apical membranes), which lie in the epithelium over Peyer's patches (see Mabbott et al. 2013). These cells sample luminal contents and transfer them to underlying immune system cells in the follicles and Peyer's patches and hence play a crucial role in intestinal immunity. Some non-epithelial cells are also present in the epithelium; these include dendritic cells, which also sample antigens by extending processes between epithelial cells into the lumen of the gut (Garrett et al. 2010) and lymphocytes. The connective tissue that lies beneath the epithelium, the lamina propria, contains a vast number of lymphocytes, in follicles, Peyer's patches, and as single cells. Mast cells and macrophages are also abundant in the lamina propria. Aging of the mucosal immune system is described in more detail later in this review.

### Changes in intestinal epithelial functions during aging

Changes in epithelial function have been examined in both humans and in animal models, but the data from different studies have again often proved inconsistent. It is generally accepted that acid secretion is not affected during non-pathological aging (see Salles 2009). Absorption of glucose in old and young individuals has been measured in a number of studies. Results from both human and animal samples, however, have produced varying results; in some cases, absorption was shown to increase with age while in others a decrease with age was described (see Drozdowski and Thomson 2006; Thomson 2009). Differences in the methods used to measure transport and the way the data are expressed (for example in relation to protein content, mucosal surface area or intestinal length) may account for the variation in these results. Changes in the enzymes of the enterocyte brush border may contribute to changes in absorption. In one study, both the expression and activities of lactase and sucrose were found to be reduced in aging rats (Lee et al. 1997). Finally, a decrease in mucus and bicarbonate secretion has been reported in aging (see Salles 2009).

Change in the barrier function of the intestinal epithelium is an important area that has been little studied during normal aging, but is of fundamental importance in maintaining the body's defences.

### Cellular changes of the aging intestinal epithelium

Age-associated changes in the architecture of the intestinal mucosa have been described in some studies, although the observations have not been consistent (see Drozdowski and Thomson 2006; Thomson 2009). For example, reduction in the height of villi and mucosal surface area in rat intestine has been reported in some studies, while other studies have reported no changes or increases (see Drozdowski and Thomson 2006). In mice, an increase in villus height with aging, but a decrease in the number of both villi and crypts with increasing age, has been measured (Martin et al. 1998). In most studies of human mucosa, however, no changes have been seen (see Thomson 2009), although such quantitative studies across the life course are clearly difficult to perform systematically on human samples.

### Changes in the populations of differentiated intestinal epithelial cells in the aging gut

Changes in the numbers or relative numbers of different types of epithelial cells during aging have been studied in humans and in mice. Increases in the numbers of some types of enteroendocrine cells in aging mouse colon (Sandstrom et al. 1998) and human duodenum (Sandstrom and El-Salhy 1999a) have been described. In the mouse duodenum, increases in some populations but decreases in others have been reported (Sandstrom and El-Salhy 2000), while no changes were reported in human rectum by the same authors (Sandstrom and El-Salhy 1999b). Once again, this suggests that there may be variability between the regions of the gut and/or species differences in age-related changes, and highlight the complexities of elucidation of age-related changes in the GI system.

Gut hormones secreted by the enteroendocrine cells play an important role in the regulation of gut functions, and some are also involved in appetite regulation. It is therefore important to establish if changes in the synthesis or release of these peptides is altered during aging. Changes in the levels of peptide hormones in gut tissues with aging have been described (El-Salhy and Sandstrom 1999).

The differentiation of the different types of the functionally distinct intestinal epithelial cells (e.g. enteroendocrine cells) depends upon activation of specific signalling pathways (see Li et al. 2011; Maloum et al. 2011; May and Kaestner 2010). The possibility that dysregulation of these pathways occurs in the aging intestinal epithelium remains to be determined.

An important aspect of aging of the epithelium is that it is exposed to potentially damaging changes in the balance of the resident microbiota and ingested materials (including pathogens) and to local inflammatory responses of the mucosal immune system. Adaptation to damage from such exposure is crucial for maintenance of the system. Changes in the rate of turnover of epithelial cells in aging human gut have been proposed as a mechanism facilitating maintenance of the mucosal epithelium in aging humans, following observations indicating increased proliferation and apoptosis in aging (Ciccocioppo et al. 2002). The turnover of the epithelium is rapid (4–5 days), thus, changes in the differentiated epithelial cell populations during aging are likely to arise from changes in the stem cells from which they develop, or possibly in the environment in which they differentiate.

### Intestinal epithelial stem cells and aging

Intestinal epithelial stem cells, which are located at the base of the crypts (Fig. 1), have been studied extensively, because mutations in these cells have been suggested as the cause of colon cancer. It has been suggested that there are two populations of intestinal epithelial stem cells: ‘deep’ or ‘quiescent’ stem cells that replicate slowly and ‘proximate’ stem cells that replicate more rapidly (Lobachevsky and Radford 2006; Radford and Lobachevsky 2006). Nearby anti-microbial-producing Paneth cells have recently been implicated in regulation of intestinal stem cells and are a key component of the stem cell niche (see Clevers and Bevins 2013). Most recently, evidence suggests that the quiescent populations are undifferentiated precursors of Paneth and hormone-producing cells that can, after injury, revert to a stem cell phenotype and thus act as a ‘clonogenic reserve’ population (see Buczacki et al. 2013; Clevers 2013). Aging of intestinal epithelial stems cells has been studied using several techniques (see Kirkwood 2004). Evidence shows that there is an increase in the incidence of mutations of mitochondrial DNA in intestinal epithelial stem cells during aging (Taylor et al. 2003). These mutations can lead to defective complexes of the respiratory chain, which are then seen in the progeny of these stem cells. Entire crypts can be formed from such stem cells, and crypt fission can lead to larger areas of affected epithelial cells (Greaves et al. 2006). Multiple respiratory chain defects have been demonstrated in some crypts of aging individuals (Greaves et al. 2010). Defects in the respiratory chain in epithelial stem cell progeny have been shown to reduce proliferation and increase apoptosis in elderly humans (Nooteboom et al. 2010). While similar mutations have been observed in both humans and mouse models, it is important to note that differences in the frequency of mutation accumulation in mice and humans has been reported (Greaves et al. 2011).

## Age-associated changes in the cells of the mucosal immune system

Mucosal defences are impaired in aging. As already outlined, the immune system of the intestinal mucosa is complex and consists of a diversity of specialised cell types; both the innate and adaptive immune systems have specialised roles in GI defences. The incidence of GI infections and inflammation increases in the elderly, and some studies have demonstrated changes in the intestinal immune system in aging animals (see Ogra 2010; Schmucker et al. 2003). Thus, although relatively few studies of aging intestinal immune system cells have been carried out, it is generally considered that mucosal immunosenescence is a feature of aging. However, as with research on other aspects of GI aging, the results from early studies of intestinal immunity are variable; and several aspects, such as immunoglobulin levels and both B and T cell functions, were not reported to be changed in aging animals (see Ogra 2010; Schmucker et al. 2003).

It has been reported that there is a decrease in the size of Peyer's patches during aging (see Fujihashi and McGhee 2004), but this has not been supported in all studies (see Schmucker et al. 2001, 2003). One change that has been established in the aging GI immune system, however, is impaired movement of immune cells both into and out of Peyer's patches. T cell migration into Peyer's patches has been reported to be decreased in aging (Ogino et al. 2004), and reduced movement of immunoblasts to the lamina propria from the Peyer's patches has also been described (see Schmucker et al. 2003). More recently, it has been reported that cytokine production and subpopulations of mucosal T cells may be reduced in aging mice (Santiago et al. 2011). Evidence for functional changes in dendritic cells (Moretto et al. 2008) and impaired maturation of M cells, resulting in reduced sampling of antigens from the lumen, has also been presented (Kobayashi et al. 2013).

## Mechanisms of cellular aging in the gut

The mechanisms that may cause death, degenerative changes or dysregulation of the different cell types of the gut during aging have been relatively little studied in comparison with other systems, such as the brain or skeletal muscle. Long-lived cells, such as enteric neurons and also differentiated smooth muscle cells are likely to accumulate damage during aging. The same may be true of some other intestinal cells, such as enteric glia and interstitial cells; however, the rate of turnover of these cells is unknown. Age-related changes to epithelial stem cells have been studied (see above), but aging of neural crest-derived neural stem cells has not been investigated.

There is good evidence that some intestinal cells develop a senescence-associated phenotype, induced by DNA damage (Jurk et al. 2012; Wang et al. 2009). The cells that display this phenotype are mucosal epithelial cells located near the crypts (Wang et al. 2009) and some neurons of the myenteric plexus (Jurk et al. 2012). However, it is possible that other intestinal cell types may display this phenotype, but only in small numbers so have not yet been detected.

The senescence-associated phenotype is associated with the production of inflammatory mediators and increased production of ROS. Myenteric neurons in older animals have been shown to have elevated ROS levels (Jurk et al. 2012; Thrasivoulou et al. 2006). Interestingly, the rate of ROS generation has been found to be lower in myenteric neurons from calorically restricted (CR) rats (Thrasivoulou et al. 2006). It has been suggested that neuronal defences against ROS-induced damage may break down during aging and disruption to neurotrophic factor support has been proposed as a mechanism contributing to neuronal aging in the gut (e.g. see Camilleri et al. 2008). This possibility has been investigated in the ENS of aging rats fed ad libitum (AL) or that were CR, in which it was found that glial cell line-derived neurotrophic factor and neurotrophin-3 reduced ROS generation in middle aged (12–15 months) CR and AL-fed rats, but this effect was only seen in CR aged (24 months) animals (Thrasivoulou et al. 2006). GDNF has also been found to protect myenteric neurons against menadione-induced cell death in intact ex vivo gut preparations (Thrasivoulou et al. 2006), and NT-3 reduces hydrogen peroxide induced neuronal cell death in dissociated cultures of isolated myenteric ganglia (Korsak et al. 2012b). GDNF and its receptors continue to be expressed in the aging rat gut, although some changes in the levels of their mRNAs were observed in 24-month AL-fed animals (Korsak et al. 2012a).

Other possible mechanisms of neuronal cell death in the aging gut have only been little studied, but there is some evidence for calcium dysregulation (see Saffrey 2013). Protein aggregation is another possible mechanism of cellular aging in the gut; α-synuclein aggregates have been demonstrated in aging gut neurons (Phillips et al. 2009).

## Aging of ‘extrinsic’ systems that interact with the gut and their impact on aging of the cells of the GI tract

The GI tract cannot be considered in isolation; there are several systems that are often considered to be extrinsic but which are closely associated with the GI tract and can have a major impact on GI aging. These systems, which have already been mentioned in the sections above, are the extrinsic innervation, the vascular system, the endocrine system and the microbiota.

### Aging of the extrinsic innervation of the gut

The gut is innervated by extrinsic autonomic and sensory nerves (see Phillips and Powley 2007). Both the parasympathetic and sympathetic divisions of the autonomic nervous system innervate the gut wall. Parasympathetic innervation is preganglionic and via the vagus nerve in the upper part of the gut and by sacral nerves in the lower parts of the intestine. Postganglionic sympathetic neurons that innervate the gut are located in the prevertebral ganglia. Sensory fibres reach the gut via the vagus nerve in the upper GI tract and from the spinal ganglia.

Aging of the autonomic and sensory systems that innervate the gut has been investigated in some studies; some signs of neurodegeneration of extrinsic neurons that supply the intestine, such as swollen nerve fibres within the gut wall, have been observed (see Phillips and Powley 2007). However, much remains to be understood about changes of extrinsic gut innervation during aging and how they impact on the cells of the GI tract.

### Aging of the intestinal vasculature

Non-pathological aging of the blood vessels within the gut wall has been very little studied but is of clear importance in our understanding of the factors that affect GI function during aging. Chen et al. (2003) reported changes in the microvessels in the villi of the rat small intestine during aging. These authors found a reduction in the volume of the microvessel network in aged rats and also presented evidence for increased vessel permeability in old animals. Age-associated disorders of the vascular system will also affect the intestinal blood vessels and may in fact cause or contribute to damage to the GI system during aging in some cases.

### Aging and the microbiota

The diversity and importance of the enteric microbiota are only now beginning to be fully understood. Gut bacteria interact in numerous ways with their host organisms. For example, they play a role in the development of the intestinal immune system (Gaboriau-Routhiau et al. 2011; Williams et al. 2006), affect GI motility (Matsumoto et al. 2012) and the function of some enteric neurons (McVey Neufeld et al. 2013), affect the brain–gut axis (Rhee et al. 2009) and profoundly influence host metabolism (Nicholson et al. 2012). Interactions between the microbiota and the host organism are continual, and disruption of the normal balance of microbial populations in the gut can result in inflammation, both locally within the gut and also more widely in the body (Garrett et al. 2010). Recent evidence has demonstrated that there are changes in the microbiota during aging (see Biagi et al. 2012; Claesson et al. 2012; Duncan and Flint 2013; Makivuokko et al. 2010; Tiihonen et al. 2010), and that these changes correlate with health status and diet (Claesson et al. 2012). This is clearly an important area for future research.

## Conclusions and future directions

Understanding aging of the GI tract and its component cells is a crucially important area of biogerontology, because of the many, varied and important functions of this organ system. Research into GI aging is entering an exciting stage, as the complex functions and interactions of different cells of the GI system, including the microbiota, are increasingly understood. Changes in individual cell types, such as enteric neurons, smooth muscle cells and intestinal epithelial stem cells during aging, are now beginning to be characterised, but it is important to understand how these changes impact on neighbouring cells, both those with which they that have specific contacts and those that are simply nearby. For example, a bystander effect may occur as a result of some GI cells developing the senescence-associated phenotype, as has been observed in vitro (Nelson et al. 2012).

The effects of diet, the microbiota and also exercise on the cells of the GI system, and the possible influences these factors may have on aging of the GI tract and its physiology are also only now beginning to be appreciated as important areas for future analysis. The complexity of cellular interactions in the GI tract and the major influences of the microbiota and diet on gut cells and their interactions mean that new approaches are needed in order to fully understand how aging influences GI functions. Furthermore, it is now clear that changes in the GI system impact upon the whole organism, so a multi-system as well as a whole-gut approach will be needed to fully understand how changes in the gut during aging affect aging of the individual. Study at the level of individual cell types alongside such studies, however, will also be needed to gain complete information about GI aging and its relationship to aging of the whole organism. Such coordinated work poses a major challenge and will require large multi-centre studies but will be essential to meet the aim of improving health and well-being in old age.
